# Paxlovid: Mechanism of Action, Synthesis, and *In Silico* Study

**DOI:** 10.1155/2022/7341493

**Published:** 2022-07-07

**Authors:** Mahrokh Marzi, Mohammad Kazem Vakil, Maryam Bahmanyar, Elham Zarenezhad

**Affiliations:** ^1^Noncommunicable Diseases Research Center, Fasa University of Medical Sciences, Fasa, Iran; ^2^Department of Internal Medicine, School of Medicine, Fasa University of Medical Science, Fasa, Iran

## Abstract

In this work, the discovery and description of PF-07321332, a major bioavailable oral SARS-CoV-2 protease inhibitor with in vitro human coronavirus antiviral activity, and excellent selection of off-target and in vivo immune profiles are reported. Various drugs and novel compound candidates for the treatment of the COVID-19 pandemic have been developed. PF-07321332 (or nirmatrelvir) is a new oral antiviral drug developed by Pfizer. In response to the pandemic, Pfizer has developed the COVID vaccine and in 2022 will launch its new major anti-SARS-Cov-2 protease inhibitor (PI). The combination of ritonavir and nirmatrelvir is under study in phase III of the clinical trial with a brand name Paxlovid. Paxlovid is an active 3Cl protease inhibitor. Paxlovid exerts its antiviral efficacy by inhibiting a necessary protease in the viral replication procedure. Proteases of coronavirus cleave several sites in the viral polyprotein where pyrrolidone was replaced by flexible glutamine. Due to the coronavirus pandemic, there is high demand for synthesis and development of this novel drug. Herein, we report the synthetic route and the mechanism of action was recently published on nirmatrelvir. Also, a comparison of the performance of two new oral antiviruses (molnupiravir and nirmatrelvir) for the treatment of COVID-19 is described. This review will be helpful for different disciplines such as biochemistry, organic chemistry, medicinal chemistry, and pharmacology.

## 1. Introduction

**Figure 1:**
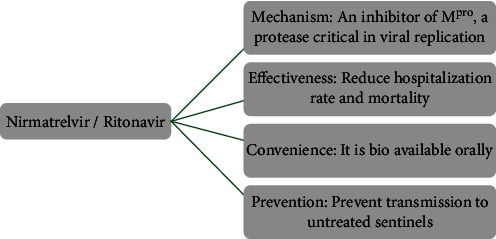
Introducing the oral form of nirmatrelvir/ritonavir as having the most promising therapeutic effect among new drugs [12].

Since the 19^th^ century, one of the great threats to public health and safety is the COVID-19 pandemic that resulted from severe acute respiratory syndrome coronavirus 2 (SARS-CoV-2) [1, 2].Until December 12, 2021, over 269 million cases and 5.3 million deaths have been demonstrated, making the COVID-19 pandemic [3]. Nirmatrelvir is a novel antiviral drug developed by Pfizer [4]. It blocks the activity of an enzyme that the virus needs to replicate. This analog of GC373 is an orally active 3Cl protease inhibitor. Corona viral proteases split multiple situations, generally after glutamine. Researchers demonstrated that the glutamine could be displaced by a hard pyrrolidone [5, 6]. 2-Pyrrolidone is a colorless liquid that can be mixed with water and other organic solvents [7]. 2-Pyrrolidone and different derivatives produced from it have various industrial uses. Nirmatrelvir acts as a covalent inhibitor that binds directly to the catalytic cysteine (Cys145) residue [8]. A phase II/III clinical trial has shown a promising drug resulting in an 89% reduction in hospitalizations, when patient is given within three days after symptom onset [9]. Recently, on November 16, 2021, the results of the phase 2/3 trial of Pfizer were presented to the U.S. Food and Drug Administration (FDA) for emergency authorization. The modification of lufotrelvir as a human clinical antiviral candidate, PF-07321332 (or nirmatrelvir), was developed [10]. Lufotrelvir is a covalent inhibitor in which the warhead is a phosphate prodrug of hydroxyketone [11]. Nirmatrelvir resulted by some modification of tripeptide protein mimetic of lufotrelvir that makes it useful for oral administration [4]. The oral form of nirmatrelvir/ritonavir offers the most promising therapeutic effect compared to these novel medicines (89% reduced risk of hospitalization or death) [12–16] (Figure 1). Nirmatrelvir/ritonavir is expected to reverse the COVID-19 pandemic [17–19].

**Figure 2:**
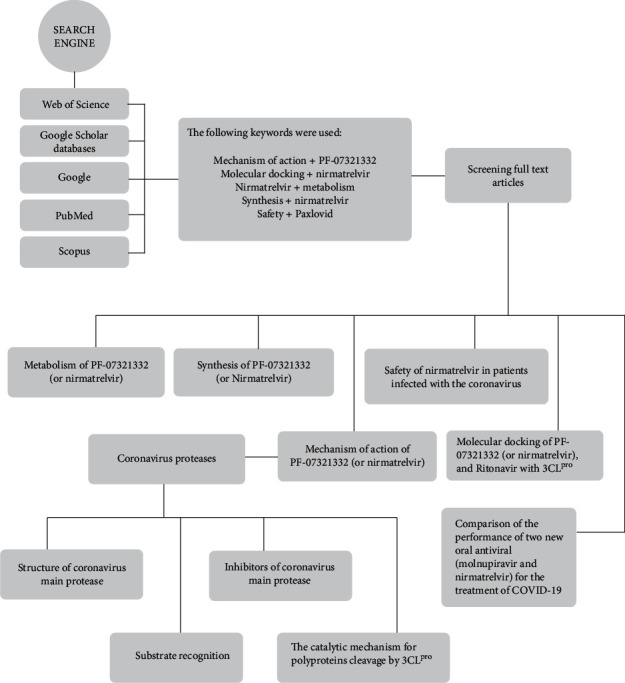
Chart related to the content provided in this review.

This review is focused on the general synthetic pathway, the mechanism of action, and molecular docking study to provide an insight for the logical synthesis of more effective nirmatrelvir as an orally antiviral candidate. The diagram of choosing publications and the content of this review is illustrated in Figure 2.

## 2. PF-07321332 (or Nirmatrelvir) Development Strategies against SARS-CoV-2

**Figure 3:**
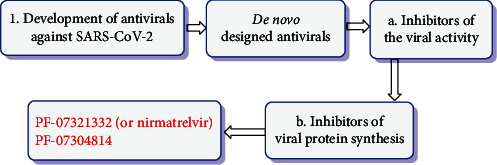
Antiviral drug development strategies against SARS-CoV-2.

From the beginning, the Pfizer Company has created a modern, useful oral antiviral for SARS-CoV-2 that behaves by controlling the infection that causes COVID-19. Nirmatrelvir, as the recent molecule (the primary protease inhibitor of SARS-CoV-2), has revealed its ability by inhibiting the replication activity of the SARS-CoV-2 virus *in vitro* and by cysteine residues, which acted as a reversible covalent inhibitor of the major protease of SARS-CoV-2. This drug is perceived as a possible treatment for future coronavirus risks. Nirmatrelvir is prescribed at the first symptom, and patients do not require to be hospitalized. This new antiviral drug is in phase one clinical trial. PF-07304814 is another compound reported by Pfizer Company with intravenous injection for hospitalized victims, and this drug is located in phase 1b multidose trial now. These antiviral drugs are being developed, as novel chemical organisms (de novo compounds) (Figure 3) [20, 21].

## 3. Synthesis of PF-07321332 (or Nirmatrelvir)

**Scheme 1:**
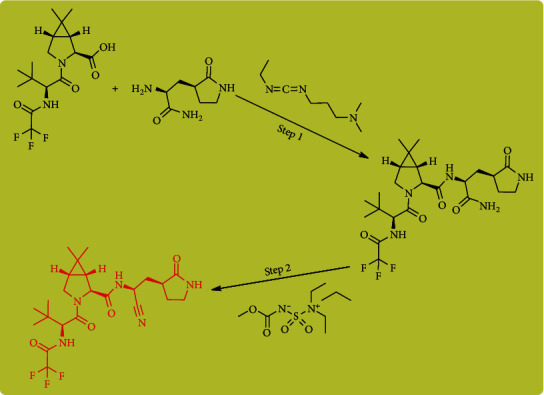
A common pathway for synthesis of PF-07321332 (or nirmatrelvir).

For the first time, Pfizer scientists published the complete particularities of the synthesis of nirmatrelvir [4]. In step (1), the water-soluble carbodiimide EDCI (as a coupling factor) paired homochiral amino amide with a synthetic homochiral amino acid. The resulting intermediate is then treated with Burgess reagent, which dehydrates the amide group to the nitrile of the product. This compound binds instantly to the catalytic cysteine (Cys145) residue of the cysteine protease enzyme (as a covalent inhibitor) (Scheme 1) [8].

### 3.1. Synthesis of *N*-[(2S)-1-({(1S)-1-Cyano-2-[(3S)-2-Oxopyrrolidin-3-yl]Ethyl}Amino)-4-Methyl-1-Oxopentan-2-yl]-4-Methoxy-1*H*-Indole-2-Carboxamide (6)

**Scheme 2:**
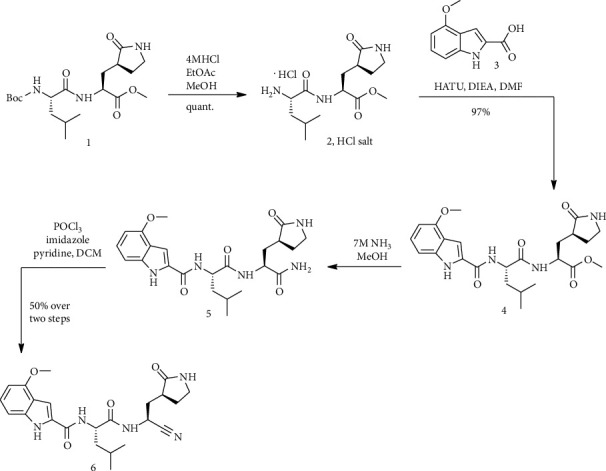
Synthesis path of compound (6).

Compound (1) [22] was stirred in a solution of hydrogen chloride in ethyl acetate and methanol for 1 h at 25°C. O-(7-azabenzotriazol-1-yl)-*N*, *N*, *N*′, *N*′*-*tetramethyluronium hexafluorophosphate (HATU) and *N*, *N*-diisopropylethylamine were added to 0°C solution of compound (2) and 4-methoxy-1*H*-indole-2-carboxylic acid in *N*, *N*-dimethylformamide. The solution was extracted after stirring at 0°C for 1.5 hours and pouring in water/ice with ethyl acetate. These organic layers with saturated aqueous sodium chloride (NaCl) solution were washed and dried by sodium sulfate [23]. Chromatography of silica gel (eluent: 1 : 10 methanol/dichloromethane) presented (4) as a yellow oil. The following was added a solution of ammonia in methanol to a resolution (4) in methanol. Following stirring, the reaction combination for 6 hours at room temperature was again added ammonia solution in methanol and stirring was continued during the night. After readding ammonia solution to methanol and stirring overnight, the last treatment was performed with ammonia solution in methanol. The reaction mixture was concentrated *in vacuo* after stirring for another day. Compound (5) was obtained after mixing the residue with the product of the same reaction performed by (4) and repeatedly dissolving the reaction mixture in ethyl acetate and concentrating. In a mixture of dichloromethane and pyridine, 1*H*-imidazole and solution (5) were cooled to −35°C (by an acetonitrile/dry ice bath). Then, phosphorus oxychloride was added over 5 minutes in a dropwise manner. The reaction was stirred for about 1.5 h (between −30°C and−20°C), then acted with HCl (hydrochloric acid), and stirred for 1 h. The resulting organic layers were concentrated *in vacuo* (after drying over sodium sulfate and filtering). The residue was blended with purified (6) from a distinctive class to produce (6) as a solid (subjected to silica gel chromatography ethyl acetate in methanol). This compound was combined with a similar reaction product performed by (5); then, it was stirred and was filtered. Compound (6) was obtained as a solid after washing with heptane and diethyl ether (Scheme 2) [4].

### 3.2. Synthesis of (1R,2S,5S)-*N*-{(2S)-1-(1,3-Benzothiazol-2-yl)-1-Oxo-3-[(3S)-2-Oxopyrrolidin-3-yl]Propan-2-yl}-3-[(4-Methoxy-1*H*-Indol-2-yl)Carbonyl]-6,6-Dimethyl-3-Azabicyclo [3.1.0] Hexane-2-Carboxamide (13)

**Scheme 3:**
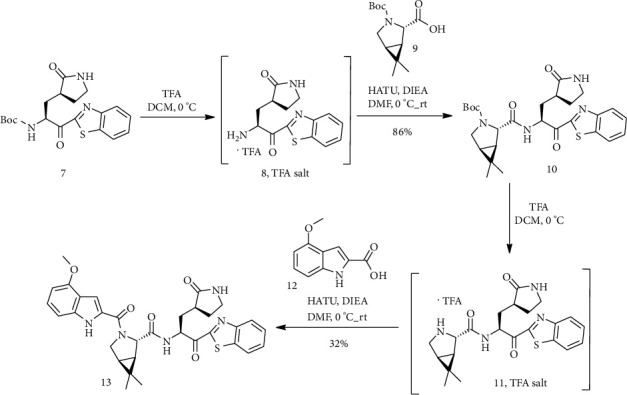
Synthesis path of compound (13).

Trifluoroacetic acid (TFA) was added to a 0°C solution of (7) [24] in dichloromethane (CH_2_Cl_2_). This mixture was stirred for 1 hour at 0°C. TFA salt of the amine (8) was cooled to 0°C and treated with *N*, *N*-diisopropylethylamine. In a different vial, a mixture of compound (9), a drop of DIES (*N*, *N*-diisopropylethylamine) in DMF (*N*, *N*-dimethylformamide), and HATU was stirred until a solution was earned (at room temperature). After adding this solution to the amine salt solution, the reaction mixture was heated at room temperature for 24 h. Compound (10) was obtained as a clear yellow oil after washing, drying, filtering, and concentrating. Then, TFA was added to a 0°C solution of (10) in CH_2_Cl_2_. Compound (11) was obtained after concentrating, cooling to 0°C, dissolving in DMF, and treating with DIEA. A solution of (12), a drop of DIEA in DMF, and HATU was added to this mixture. After 2 hours, the reaction combination was diluted with EtOAc to form compound (13) (Scheme 3) [4].

### 3.3. Synthesis of (1R,2S,5S)-*N*-{(2S)-1-(1,3-Benzothiazol-2-yl)-1-Oxo-3-[(3S)-2-Oxopyrrolidin-3-yl]Propan-2-yl}-6,6-Dimethyl-3-[*N*-(Methylsulfonyl)-L-Valyl]-3-Azabicyclo [3.1.0] Hexane-2 Carboxamide (19)

**Scheme 4:**
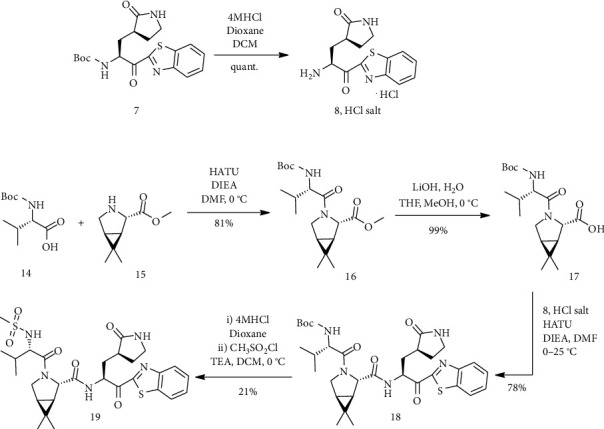
Synthesis path of compound (19).

Solution (7) in CH_2_Cl_2_ was treated with a solution of hydrochloride in 1,4-dioxane and then using ethyl acetate. The reaction mixture was stirred at room temperature, and then, it was condensed *in vacuo*. The remaining trituration with diethyl ether (Et_2_O) obtained a compound of bright-yellow solid (the HCl salt of (8)) that was utilized without more purification in the next step. In the following, a 0°C mixture of (14), (15), and DMF was treated with HATU. Then, stirring was continued after the addition of DIEA (for 2 h at 0°C). The incorporated organic layers were concentrated *in vacuo* and were prepared (16) as gum. Aqueous LiOH (lithium hydroxide solution) was poured in the form dropwise into a 0°C solution of (16) in a mixture of methanol (CH_3_OH) and tetrahydrofuran. Aqueous LiOH solution was added to the stirred reaction mixture for 2 h at 0°C and 4 h at room temperature, and then, stirring was continued for 15 minutes. After repeating the previous operation, compound (17) was obtained as an off-white solid. To a 0 °C solution of (17) , HATU and N,N-diisopropylethylamine in N,N-dimethylformamide were added (8), hydrochloride salt. DIEA was added, and the reaction mixture was stirred and made warm to room temperature overnight. Compound (18) was provided as oil after dilution by ethyl acetate. To a solution of compound (18) in CH_2_Cl_2_, a solution of HCl in 1,4-dioxane was added and the reaction mixture was stirred for 2 h at room temperature. Stirring was continued, and then, the reaction mixture was concentrated. To this mixture, triethylamine (Et_3_N) and methanesulfonyl chloride were added. Then, compound (19) was produced as a white solid (Scheme 4) [4].

### 3.4. Synthesis of (1R,2S,5S)-*N*-{(2S)-1-(1,3-Benzothiazol-2-yl)-1-Oxo-3-[(3S)-2-Oxopyrrolidin-3-yl]Propan-2-yl}-6,6-Dimethyl-3-[*N*-(Trifluoroacetyl)-L-Valyl]-3-Azabicyclo [3.1.0] Hexane-2-Carboxamide (23)

**Scheme 5:**
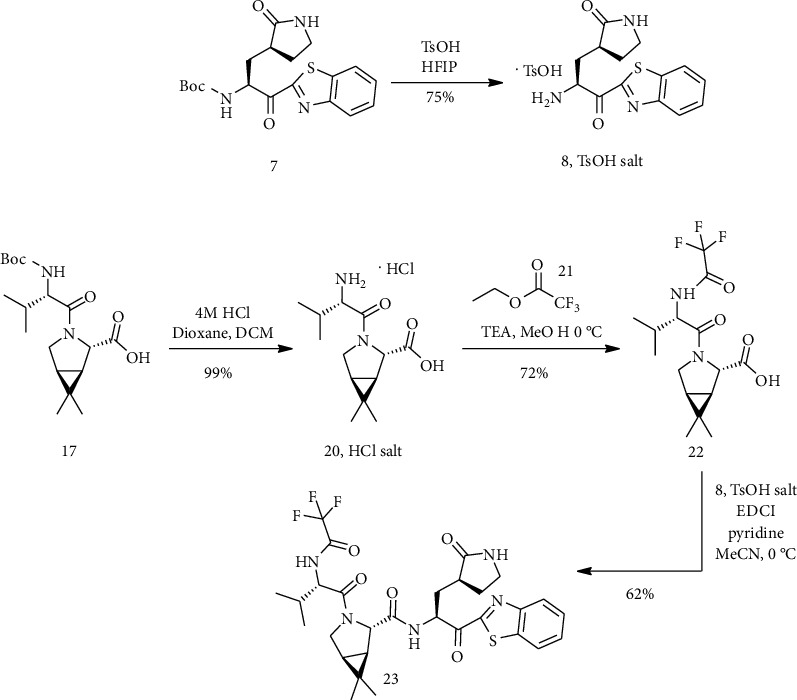
Synthesis path of compound (23).

A solution of compound (7) in hexafluoroisopropanol (HFIP) was cooled in an ice bath and was treated with para-toluenesulfonic acid. After the reaction mixture had been stirred at room temperature it was concentrated; trituration of the residue with ethyl acetate provided (8) para-toluenesulfonate salt. Then, a solution of HCl in 1,4-dioxane is added to the solution (17) in CH_2_Cl_2_ and mixed. A white solid (20) was obtained by removing the solvent. To a 0°C solution of (20) in CH_3_OH, Et_3_N was added, and then, the reaction mixture was warmed. After adding compound (21) to the reaction mixture, compound (22) was produced as a white solid. In the next step, EDCI was added to combination (8), para-toluenesulfonate salt, and a solution of (22) in anhydrous acetonitrile at 0°C. Pyridine was added dropwise to the stirring reaction mixture. Filtration acquired (23) as a white solid (Scheme 5) [4].

### 3.5. Synthesis of PF-07321332 or Nirmatrelvir (33)

**Scheme 6:**
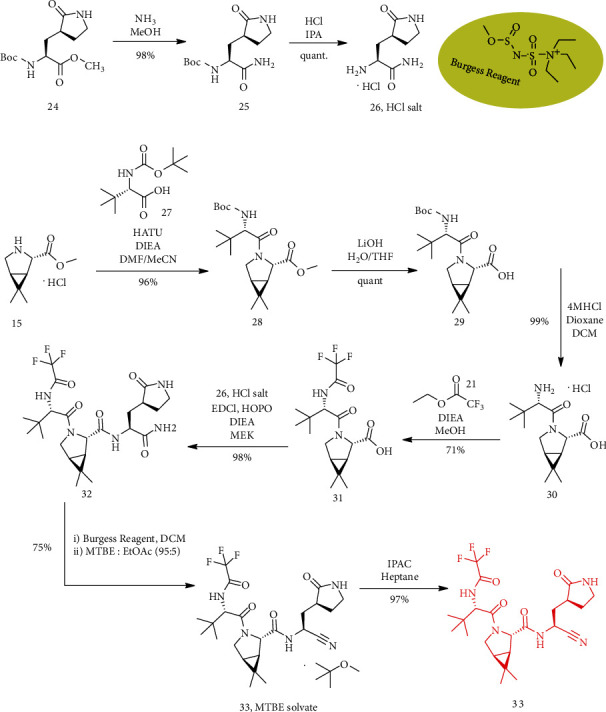
Synthesis path of compound (33).

Compound (25) was obtained as a yellow solid by adding ammonia solution in methanol to compound (24). The solution of HCl in isopropanol was added to a 0°C solution of (25) in isopropanol. Then, it was concentrated to give the HCl salt of (26). *O*HATU was added to a 0°C solution of (15) and (27) in a mixture of DMF and acetonitrile (CH_3_CN), followed by dropwise addition of DIEA. Then, compound (28) as a colorless oil was provided. To a solution of (28) in tetrahydrofuran, water and lithium hydroxide (LiOH) were added. Compound (29) was produced after washing, drying, and concentrating. To a solution of (29) in CH_2_Cl_2_, a solution of hydrochloride in 1,4-dioxane was added and the reaction mixture was stirred for 16 h at room temperature. By removing the solvents, HCl salt of (30) was generated. To a solution of the HCl salt of (30) in CH_3_OH, Et_3_N was added, followed by compound (21); then, the reaction mixture was warmed to 50°C and was stirred. The incorporated organic layers were washed with sodium chloride (NaCl) solution, and compound (31) was obtained as a white solid. To a solution of (31) and the HCl salt of (26) in butan-2-one, 2-hydroxypyridine 1-oxide was added and the mixture reaction was cooled (to 0°C). Then, DIEA and 1-[3-(dimethylamino) propyl]-3-ethylcarbodiimide hydrochloride (EDCI) were added and compound (32) was provided. For preparing compound (33) (MTBE solvate), a solution of (32) in CH_2_Cl_2_ to methyl *N*-(triethylammoniosulfonyl) carbamate and inner salt (Burgess reagent) was added. Compound (33) (anhydrous MTBE solvate) was charged to a reactor at 350 rpm. Stirring was continued overnight with the addition of heptane and isopropyl acetate (IPAC). Compound (33) was acquired as a white crystalline solid (Scheme 6) [4].

During the discussion of synthesis methods, different reaction parameters (temperature, solvent, etc.) affected selectivity and efficiency (Table 1) [14].

## 4. Mechanism of Action of PF-07321332 (or Nirmatrelvir)

For the treatment of COVID-19, the antiviral medicine of nirmatrelvir reacts as an orally active inhibitor of 3CL protease. The blend of ritonavir with nirmatrelvir is in phase III trials [25–29], and under the brand name, Paxlovid is anticipated to be sold [30]. By cytochrome enzymes, ritonavir dwindles the metabolism of nirmatrelvir to preserve premier concentrations of the principal drug [31].

### 4.1. Coronavirus Proteases

**Figure 4:**
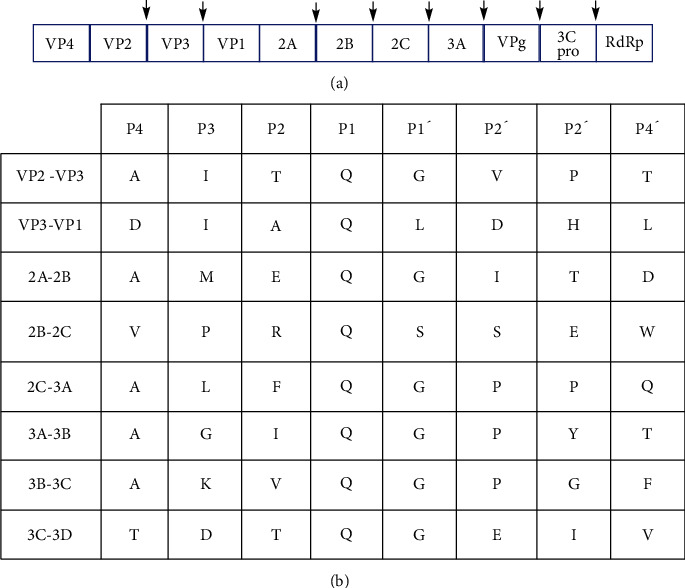
Enterovirus 3C protease (3C^pro^). (a) Schematic exhibition of the enterovirus polyprotein, with 3C^pro^ division sites displayed by arrowheads. (b) Amino acid succession of polyprotein division sites. The protease splits among P1-P1′.

The severe acute respiratory syndrome coronavirus 2 is the cause of the coronavirus epidemic [32]. By mid-2021, more than 170 million confirmed infections had been announced worldwide. In the 21st century, the 2019 epidemic is the third outbreak of coronavirus [33, 34]. Different types of the syndrome such as the severe acute respiratory syndrome coronavirus (SARS-CoV), Middle East respiratory syndrome (MERS-CoV), and SARS-CoV-2 depend on the *Betacoronavirus* genus (the *Coronaviridae* family). Coronaviruses encoded papain-like protease (PL^pro^) (also known as nsp3) and main protease (M^pro^) (also known as 3CL^pro^ or nsp5) to split the viral polyprotein [35]. M^pro^ has a notable role in polyprotein processing as a more tractable therapeutic objective (Figures 4(a) and 4(b)) [36]. M^pro^ from SARS-CoV-1 and SARS-CoV-2 has an excellent general sequence identity [36, 37].

#### 4.1.1. Structure of the Coronavirus Main Protease

M^pro^ is a residual protease of 306 with a catalytic binary consisting of histidine and cysteine. Active-state M^pro^ is a dimer, in which the two monomers are arranged orthogonally (biologically) [38]. Each monomer consists of three areas; two anti-*β* barrels parallel to the fold-like trypsin-like serine proteases are built by areas I and areas II together. Area III includes five *α*-helices which are attached by the linker to area II. Dimerization of the protomers occurs at the N-terminus(N-finger) located between domains II and III [39, 40].

#### 4.1.2. Substrate Recognition

In addition to the catalytic binary consisting of H47 and C145, M^pro^ contains a molecule of water buried in the active site that replaces the aspartate residue, which is usually combined with histidine and cysteine in the conventional catalytic triplet of other proteases [36, 41]. A multistep mechanism pursues the catalytic process, in which H47 removes the catalytic cysteine from the proton and produces a nucleophilic thiol that attacks the carbonyl carbon of the scissor bond and forms a quadrilateral intermediate. The intermediate falls and remains an acylated intermediate. The thiol side chain is regenerated by histidine, which activates a water molecule and hydrolyzes the intermediate [42, 43]. As shown in Figure 2, M^pro^ at 11 sites splits the viral polyproteins (pp1a and pp1ab). The amino acid sequence is varied at the sites of cleavage, except in P1, which is always glutamine, and P2, which is preferred for leucine. The P3 situation can insert hydrophilic and hydrophobic and residues, and for amino acids with tiny side chains, P4 has superiority. M^pro^ identifies substrates spanning P4-P1 [44].

#### 4.1.3. Inhibitors of the Coronavirus Main Protease

Inhibitors of −2 M^pro^ and SARS-CoV-1 have been evaluated widely [37, 42, 45, 46]. Small molecules and peptidomimetics containing Michael receptors, for example, ketones and aldehydes, form a significant part of the designed inhibitors. The P1*γ*-lactam group as a glutamine mimetic is a standard feature of these inhibitors. PF-07321332 (or nirmatrelvir) and PF-07304814 (two dipeptidyl inhibitors) are arriving phase I clinical trials (NCT04756531 and NCT04535167) [10, 11]. PF-07304814, the prodrug form of PF-00835231, contains a phosphonate group for better solubility that gets cleaved by alkaline phosphatase enzymes in tissue. Nirmatrelvir was supposed to dominate the intravenous delivery method for PF-07304814.

#### 4.1.4. The Catalytic Mechanism for Polyprotein Cleavage by 3CL^pro^

**Scheme 7:**
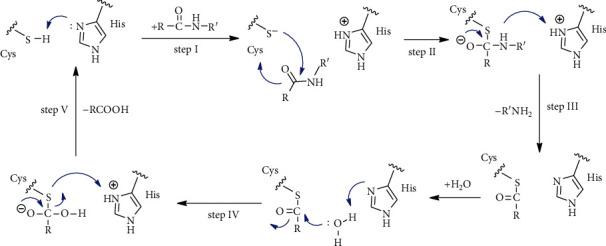
Schematic representation of the catalytic mechanism for the division of polyproteins by 3CL^pro^.

By inferring the structure and analysis of the SARS-CoV 3CL^pro^ [42] autolytic section (automated processing), a universal nucleophilic reaction mechanism for polyprotein cleavage has been proposed by 3CL^pro^ [43]. In the first step, the catalytic double Cys-thiol is released from the proton with the help of adjacent histidine. In the next step, the anionic sulfur strikes the carbonyl carbon of the scissile amide bond. A peptide product is then dropped with an amine terminus; on the other hand, the histidine recovers the deprotonated form (step III). Finally, the thioester produced to release carboxylic acid (IV) is hydrolyzed and the catalytic binary returns to its original state, preparing it for the next proteolytic cycle (Scheme 7).

## 5. Safety of Nirmatrelvir in Patients Infected with the Coronavirus

**Figure 5:**
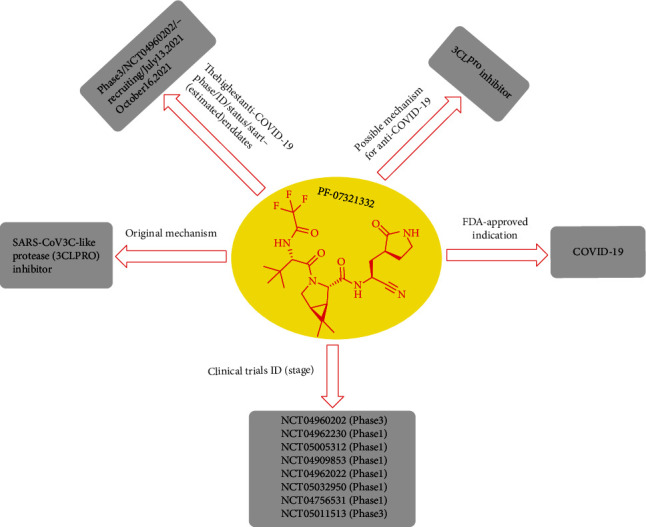
Diagram related to general specification nirmatrelvir.

Pfizer Company reports that the oral antiviral drug nirmatrelvir notably reduces the acceptance and mortality of patients with COVID-19 at higher risk for severe disease than placebo. Brief analysis of the data obtained in phases II and III showed that 1219 adults had registered by September 29, 2021. The results obtained among participants who were treated for 3 days after the onset of symptoms of COVID-19 showed that the risk of hospitalization for COVID-19 or death in the nirmatrelvir group was 89% lower than that in the placebo group. In the study, trial participants were randomly selected 1 : 1, half of whom received nirmatrelvir and the other half orally placebo for five days every 12 hours. Among those treated within three days of the onset of symptoms, 0.8% (3.389) of patients who received nirmatrelvir were hospitalized until the 28th day after randomization without death. In comparison, 7% (27.385) of placebo patients were hospitalized with seven deaths. A similar decrease was observed in patients treated within five days of the onset of symptoms, with 1% (6.607) in the nirmatrelvir group by day 28 (without death) and 6.7% (41.612) in the placebo group (10 deaths). In general, by day 28, no casualties were informed among patients receiving nirmatrelvir, while 10 (1.6%) in the placebo group lost their lives (Figure 5) [25, 30, 47, 48]. In another study, high confidence was obtained in the selection of the 300 mg nirmatrelvir diet in combination with the 100 mg ritonavir as BID over a 5-day term for phase 2/3 clinical trials in patients with COVID-19 [49]. Older people are at higher risk for severe complications from COVID-19. Explanation of PIMs before COVID-19 infection can increase the relation of older people that can safely be given nirmatrelvir-ritonavir, in addition to the ordinary advantages seen with drug administration [50–52].

## 6. Molecular Docking of PF-07321332 (or Nirmatrelvir) and Ritonavir with 3CL^pro^

The *molecular docking* studies can be applied to model the interaction among a specific protein and small molecule at the atomic level, which helps us to determine the behavior of small molecules in the binding site of the target protein [53, 54]. In a study, the binding of PF-07321332 and ritonavir to SARS-CoV-2 3CL^pro^ was investigated by molecular dynamic (MD) simulation and molecular mechanics Poisson-Boltzmann surface area (MMPBSA) calculation [27]. *α*-Ketoamide and PF-07321332 (NCT04756531) molecules, which in a particular bind to and prevent SARS-CoV-2 3CL^pro^, could be promising alternatives for fighting the epidemic [55]. A relative binding state investigation of PF-07321332 and ritonavir may supply an overview of the design of rational drugs by modifying inhibitors based on residues in the enzyme-active site. Molecular docking of PF-07321332 and ritonavir to 3CL^pro^ was performed to resolve the mechanism of binding these molecules. By the docking score and the best ligand binding mode, the best docking solutions for PF-07321332 and ritonavir were chosen. To realize the binding mechanism of PF-07321332, combined with the antiretroviral drug ritonavir to 3CL^pro^ were done MD simulations for 100 ns. The RMSD of 3CL^pro^ was shown to be more stable than with ritonavir associated with either PF-07321332. Unlike other systems, 3CL^pro^ in complex with ritonavir significantly deviates between 88 and 95 ns from the simulation. PF-07321332 ligand with 3CL^pro^ is more stable than ritonavir because the ligand was later found to deviate during the simulation. The latest clinical trials have also shown that these antiretroviral drugs are unsuccessful because they do not significantly accelerate clinical improvement in severe COVID-19 patients [56]. RMSF 3CL^pro^ was analyzed in all two complexes to evaluate the effect of ligand binding on protein residues. Amino acid residues 45–65 with ritonavir displayed more significant fluctuations than apo-form. These oscillation zones are mainly composed of junctional residues that are connected by a hydrogen bonding network and are involved in the formation of the catalytic pair between Cys145 and His41 [57–59]. 3CL^pro^-PF in the RMSF of the protein revealed the same tendency as its apo-form. PF-07321332 displayed more powerful interactions with 3CL^pro^ (in the interaction analysis), and these interactions during the simulation stayed intact because between the interacting groups, the minimum distance remained without change. The interactions (Cys145–H9 and Cys145–O2) in 3CL^pro^-PF during the simulation stayed intact. This indicated that PF-07321332 is firmly attached to 3CL^pro^ [60]. Gly143 SARS-CoV-2 3CL^pro^ has been reported as the most desirable residue for forming hydrogen bonds with ligands, followed by Cys145, His163, and Glu166 (Table 2) [61]. In similar research, the recognized compounds bind to the similar binding site but the resistance of the complex is less than the one of PF-07321332 [62, 63]. Given the current epidemic position, it is essential to find a potent candidate drug with desirable binding affinity.

**Figure 6:**
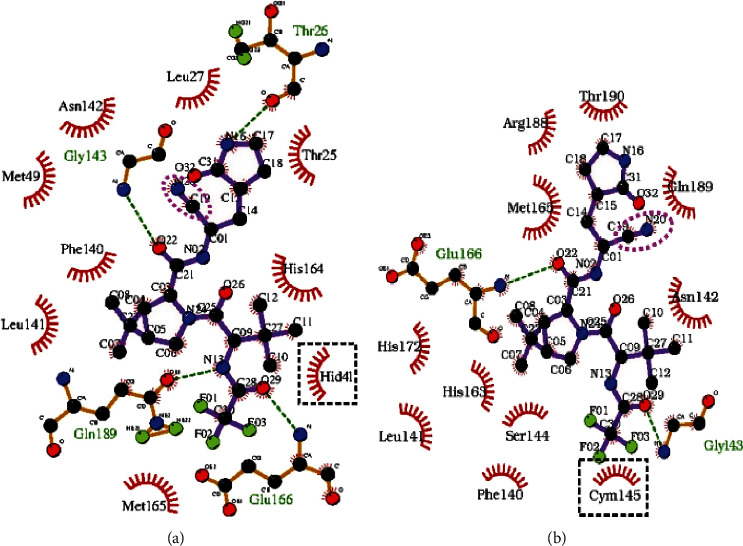
Two-dimensional display of the best docking poses at the connection achieved with LIGPLOT [68]. The nitrile group is a circular magenta color. The hydrogen bonds with dashed green lines and the binary residues are shown with black dashed squares. (a) Dyad in the neutral condition. (b) Dyad in zwitterionic condition.

Macchiagodena et al. [64] have investigated the noncovalent interaction between PF-07321332 and SARS-CoV-2 main protease at the atomic level using a computational approach based on extensive molecular dynamics simulations with explicit solvent. This drug, in mixture with ritonavir, depends on the electrophilic invasion of a nitrile cap to the catalytic cysteine of the protease. Nonbonding interactions between the residues of the binding pocket and the inhibitor and with water molecules on the protein level have been determined by the two possible protonation states and two distinct force fields of the major protease catalytic binary HIS41-CYS145. When the catalytic binary is in the impartial state, the noncovalent bond is probably stronger. Simulation (MD) appears to hold up a two-step inhibitory mechanism: (a) adding noncovalently paired to a neutral form and (b) thiolate-imidazolium formation and ligand displacement to finalize the electrophilic attack (Figure 6) [64].

PF-07321332 reacts with a cysteine residue at the protease binding site to stop viral repetition in cells (as a 3CL^pro^ inhibitor). This cysteine plays an important part in the activity of 3CL^pro^ coronaviruses. Thus, inhibition of activity prevents the encoding of the viral genome of functional NSPs and ultimately suppresses virus replication [44, 65]. PF-07321332, with its nitrile group, acts as a polypeptide covalent inhibitor. Peptide nitriles react with catalytic cysteine as site-specific reversible inhibitors to form sulfides. This inhibitor provides a g-lactam ring, a usual attribute of most covalent inhibitors developed against SARS-CoV-2 3CL^pro^, which utilizes the selectivity of glutamine residues in the cleft multiprotein equivalent site [66, 67].

## 7. Comparison of the Performance of Two New Oral Antiviruses (Molnupiravir and Nirmatrelvir) for the Treatment of COVID-19

Although the coronavirus disease epidemic has not been fully controlled but significant advances have been made in COVID-19 studies [1, 2] and antiviral drugs have displayed good therapeutic results against COVID-19, an easy oral antiviral drug for COVID-19 has not yet been expanded. Molnupiravir is a small molecular prodrug of the nucleoside derivative *N*-hydroxycytidine (NHC). NHC triphosphate attacks viral RNA polymerase and has been demonstrated to prevent the repetition of several RNA viruses containing SARS-CoV-2 [69, 70]. Considerable clinical advantages have been shown in a vast phase 3 clinical trial for COVID-19 patients distributed orally with molnupiravir [71]. This is the first accepted oral antiviral factor for the treatment of COVID-19. MTP can be employed by the RNA-dependent RNA polymerase (RdRp) of SARS-CoV-2 instead of cytidine triphosphate (CTP) or uracil triphosphate (UTP) as a substrate. First, RdRp probably develops the combination of molnupiravir instead of C or U in the synthesis of positive-strand genomic RNA that acts as a template for the synthesis of negative-strand genomic RNA and subgenomic RNA. In the next step, the negative-strand genomic RNA comprising molnupiravir can be applied as a sample for the synthesis of positive-strand genomic RNA or positive-stranded subgenomic mRNA [72]. Paxlovid is composed of the protease inhibitor PF-07321332 (designed specifically for the protease SARS-CoV-2-3CL) and ritonavir (used in HIV therapy). The purpose of ritonavir is to increase the effectiveness of an oral protease inhibitor by stopping the rapid metabolism of PF-07321332 using liver enzymes, so maintaining a sufficient circulating concentration of PF-07321332 to inactivate the virus [73]. PF-07321332 is responsible for the cleavage of precursor proteins to structural proteins and enzymes that act on virus replication and maturation by inhibiting viral proteolysis mediated by SARS-CoV-2-3CL protease [73–76].

In the other study, nirmatrelvir (PF-07321332), and other clinically relevant SARS-CoV-2 antivirals, were tested against a panel of SARS-CoV-2 variants, including the novel Omicron variant, in live-virus antiviral assays. [77]. Rosales [77] confirmed that nirmatrelvir and other clinically related antiviral drugs all retain their activity against all types tested, including Omicron.

Li et al. [78] have shown that molnupiravir and nirmatrelvir strongly inhibit SARS-CoV-2 Omicron infection. A combination of molnupiravir and nirmatrelvir applied synergistic antiviral property. Remarkably, there are delicate differences in antiviral response patterns between the Omicron, WT, and Delta variants, also between cell lines and organoid patterns. However, these discoveries support the use of molnupiravir and nirmatrelvir in the treatment of patients with Omicron.

Omicron-type acute respiratory distress syndrome 2 (SARS-CoV-2) is currently the most prevalent type in the United States, and its numerous mutations have invalidated some previously authorized treatments [79]. The US National Institutes of Health (NIH) has published a list of drugs that should now be investigated for use (in the treatment of outpatients). Nirmatrelvir with ritonavir (Paxlovid) as one of these drugs is understood to be clinically effective and prioritized for ease of administration. For the treatment of mild to moderate COVID-19, it is essential to understand the key principles about recommended treatments. Nirmatrelvir, the antiviral active ingredient in Paxlovid, inhibits the main SARS-CoV-2 protease, Mpro, thus preventing the virus from replicating [80, 81]. Ritonavir inhibits the metabolism of nirmatrelvir by CYP3A, thereby increasing the plasma concentration of nirmatrelvir and by itself having no activity against SARS-CoV-2. Ritonavir-induced drug interactions may occur, and renal and hepatic impairment may limit the use of Paxlovid in some patients. Ritonavir is known for its use as an HIV-1 protease inhibitor, and the potential for antiretroviral resistance must be confirmed by the prescriber. Amid the limited supply of new treatments for mild to moderate COVID-19 outpatient treatment, physicians face new challenges, especially among those practicing in military medical centers abroad [82]. Although prescribers may not be familiar with these drugs, their proper use requires careful examination of the patient's symptoms and familiarity with the characteristics of the side effects of each drug. Given these complexities, a step-by-step guide is provided here to assist physicians in managing outpatients with mild to moderate COVID-19 by nirmatrelvir with ritonavir (Paxlovid) as one of these effective drugs.

## 8. Conclusions

Nowadays, the COVID-19 pandemic is one of the biggest challenges which is the leading cause of death in many countries in the globe. Therefore, the discovery and development of new drugs for novel coronavirus (CoV) are essential. This review is focused on summarizing recent research on nirmatrelvir. It will aid researchers, organic chemists, medicinal chemists, pharmacologists, etc., in the discovery and the synthesis of the new effective antiviral compounds. Nirmatrelvir as an orally antiviral agent displayed a promising combination for nonhospitalized patients with COVID-19.

## Figures and Tables

**Table 1:** The influence of various reaction parameters on the selectivity and efficiency of synthetic compounds.

Compounds	Temperature	Time	Solvent	pH	Yield	Ref.
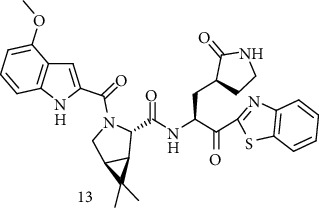	4°C	18 h	(100% DMSO) for a molar ratio of approximately 1 : 7	6.00	18.4 mg, 30.7 *μ*mol, 32%	[14]
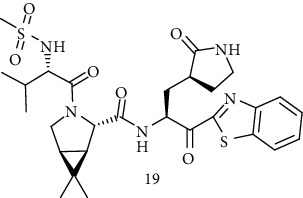	4°C	18 h	(100% DMSO) for a molar ratio of approximately 1 : 5	7.00	18 mg, 30 *μ*mol, 21%	[14]
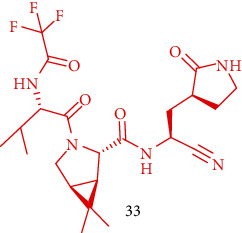	4°C	24 h	—	6.00	160.93 g, 322 mmol, 97%	[14]

**Table 2:** Residue and ligand atom interactions with the bond type and energy in 3CL^pro^ complexes.

Complex	Residue	Ligand atom	Bond type	Energy
3CL^pro^-PF	Cys145	O_2_	Hydrogen	−5.04
	Cys145	H_9_	Hydrogen	−5.04
	Glu166	O_3_	Hydrogen	−4.41
	Gln189	H_2_O	Hydrogen	−3.01

3CL^pro^-rit	His41	H_6_	Arene	−3.0
	Gly143	O_3_	Hydrogen	−2.2
	Pro168	Thiozyl group	Arene	−0.8
	Thr190	S_2_	Hydrogen	−0.8

## Data Availability

The datasets used and analyzed during the current study are available from the corresponding author upon reasonable request.

## Abbreviations

BID: Nirmatrelvir/ritonavir twice daily
3CL^pro^: 3-Chymotrypsin-like protease
CH_3_CN: Acetonitrile
CH_3_OH: Methanol
CTP: Cytidine triphosphate
Cys145: Cysteine
DCM, CH_2_Cl_2_: Dichloromethane
DIEA: *N*,*N*-Diisopropylethylamine, Hunig's base
DMF: *N*, *N*-Dimethylformamide
EDC, EDAC or EDCI: 1-Ethyl-3-(3-dimethylaminopropyl)carbodiimide
Et_3_N: Triethylamine
Et_2_O: Diethyl ether
EtOAc: Ethyl acetate
HATU: O-(7-Azabenzotriazol-1-yl)-*N*,*N*,*N*,*N*-tetramethyluronium hexafluorophosphate
HCl: Hydrogen chloride
HFIP: Hexafluoroisopropanol
His: Histidine
HOPO: 2-Hydroxypyridine 1-oxide
IPA: Isopropanol
IPAC: Isopropyl acetate
LiOH: Lithium hydroxide
MD: Molecular dynamic
MEK: Methyl ethyl ketone, butan-2-one
MMPBSA: Molecular mechanics Poisson-Boltzmann surface area
M^pro^: Main protease
MTBE: Methyl tert-butyl ether
NaCl: Sodium chloride
NHC: *N*-Hydroxycytidine
NSPs: Nonstructural proteins
PI: Protease inhibitor
PIMs: Potentially inappropriate medications
PL^pro^: Papain-like protease
RdRp: RNA-dependent RNA polymerase
RMSD: Root mean square deviation
SARS-CoV-2: Severe acute respiratory syndrome coronavirus 2
TFA: Trifluoroacetic acid
TsOH: Para-toluenesulfonate
UTP: Uracil triphosphate
WT: Wild type.
